# Changes in alcohol consumption during the COVID-19 pandemic: a longitudinal cohort study using smart-breathalyzer data

**DOI:** 10.1038/s41598-024-53757-y

**Published:** 2024-02-08

**Authors:** Parker D. Houston, Eric Vittinghoff, Gregory M. Marcus

**Affiliations:** 1 School of Medicine, University of California, San Francisco, USA; 2 Department of Epidemiology and Biostatistics, University of California, San Francisco, USA; 3 Division of Cardiology, University of California, 505 Parnassus Avenue, M1180B, San Francisco, CA 94143 USA

**Keywords:** Epidemiology, Cardiology, Public health

## Abstract

Previous studies relying on alcohol sales, alcohol-related injuries, and surveys have suggested that alcohol consumption increased during the COVID-19 pandemic. We sought to leverage over 1 million Breath Alcohol Concentration (BrAC) measurements from Bluetooth-enabled breathalyzers to conduct an objective and longitudinal assessment of alcohol use during the pandemic. Serial BrAC measurements revealed a decrease in drinking between January 1, 2020 and March 30, 2020, an increase between March 30, 2020 and May 25, 2020, a statistically insignificant decrease between May 25, 2020 and January 1, 2021, and an increase again between January 1, 2021 and June 4, 2021. No statistically significant relationships between shelter-in-place orders and alcohol consumption were detected. These findings demonstrate the complex relationship between the pandemic and alcohol consumption patterns, providing insights that may be relevant to the use of this commonly consumed substance with implications relevant to long-term effects from the patterns observed.

## Introduction

The World Health Organization’s declaration of the severe acute respiratory syndrome coronavirus 2 (SARS-CoV-2, or COVID-19) global pandemic triggered several measures across the world to reduce the spread of the virus. The initial surge of SAR-CoV-2 within the United States led to most states adopting shelter-in-place (SIP) orders restricting large gatherings and in-restaurant dining, and closure of nonessential businesses. By April 6, 2020, forty-two states plus Washington, D.C. had statewide SIP orders in place^1^. Studies from the early pandemic period reported increases in psychological distress, including symptoms of anxiety and depression across many different populations in the United States^2–4^.

Alcohol is one of the leading behavioral causes of global disease burden and mortality^5^. Heavy alcohol consumption and binge drinking have been associated with an increased risk of violence, poverty, several cardiovascular diseases, cancer, and sexually transmitted diseases^6–9^. It has long been shown that both acute and chronic forms of stress can increase the risk of binge drinking among adults^10,11^. Additionally, mental health disorders, including depression and anxiety disorders, have been associated with increased alcohol-related behaviors such as binge drinking and alcohol use disorders^12–15^. Thus, new and life-disrupting events like those occurring with the COVID-19 pandemic might be expected to lead to increasing alcohol consumption.

Alcohol-related deaths^16^ and alcohol sales^17^ increased during the early months of the pandemic, coinciding with self-reports of more alcohol consumption among some^11^. However, given the stigma surrounding alcohol use as well as evidence that self-reports may not reliably reflect alcohol consumption^18,19^, it remains unclear whether and to what extent alcohol consumption patterns within the US actually changed with the pandemic. In addition, the timing of such changes and whether they have been sustained have yet to be elucidated.

Lay-public use of commercially available personal breathalyzers has grown throughout the world. Within the past few years, the use of Bluetooth technology has been incorporated into these devices, enhancing their accessibility. These smart breathalyzers are highly accurate devices^20^ that automatically track, store, and time-stamp every breath alcohol (BrAC) measurement. While breathalyzers were traditionally used to help monitor alcohol content to avoid injury while driving or working, recent advances in these devices may broaden their application for personal use. Leveraging Bluetooth-enabled breathalyzer data to study alcohol use behaviors may offer several advantages. Because the data collected from these devices requires no additional effort beyond the use of the breathalyzer, the information gleaned from these devices reflects real-world and real-time behaviors. Many previous studies reporting on alcohol use behavior have relied on self-report, potentially introducing bias and inaccurate recall, whereas breathalyzer data provides a quantified and objective assessment^19,21^. Additionally, these data reveal real-time and repeated tracking, rather than relying on an average (generally requiring the participant to both remember amounts and to do the arithmetic before answering) or general estimate over some time period. We sought to interrogate the trends of alcohol use during the COVID-19 pandemic by leveraging over one million BrAC measurements from more than 25,000 persons using smart-breathalyzers in the course of their daily lives.

## Results

**Figure 1:**
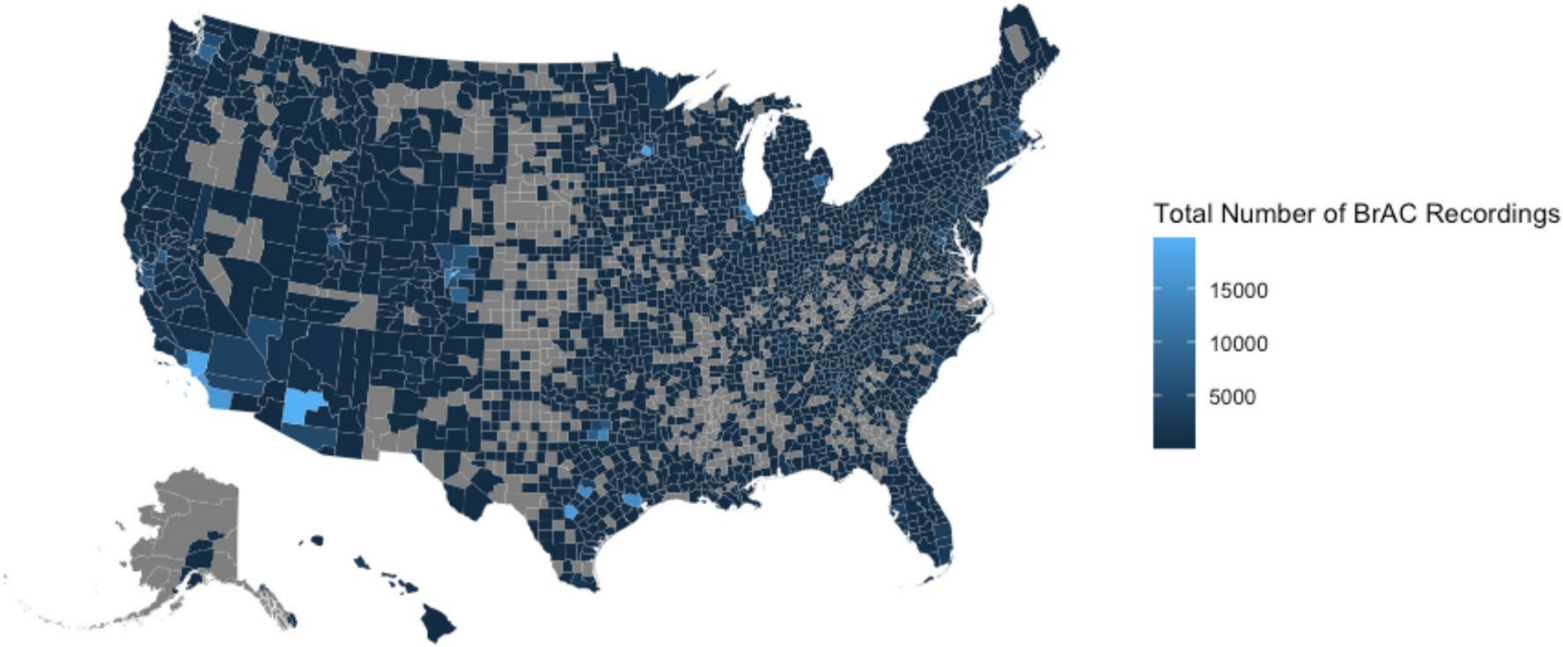
Total Number of BrAC observations made, grouped by United States counties. The value within each county on the U.S. map represents the total number of BrAC recordings measured within the county between January 1, 2019, and June 4, 2021. Grey represents counties where no BrAC measurements were made. This map was generated by the studio authors using R (Vienna, Austria) with the urbnmapr package (https://urbaninstitute.github.io/urbnmapr/).

**Figure 2:**
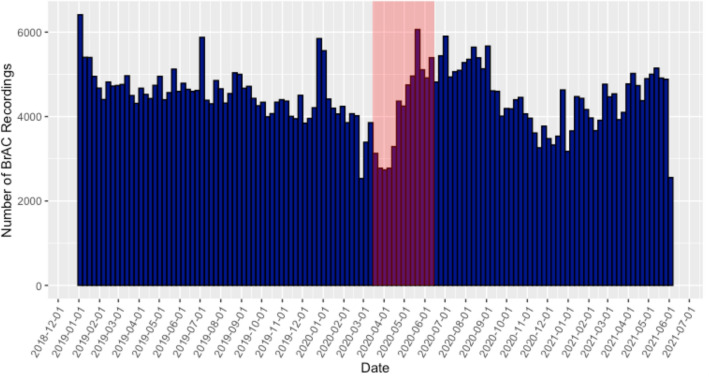
Total number of BrAC recordings made by calendar week. Each blue box represents the total number of BrAC measurements recorded within each calendar week between January 1, 2019, and June 4, 2021. The area shaded red represents the dates between March 15, 2020 and June 15, 2020 where “Mandatory for All Individuals” Shelter-in-Place orders were placed in at least one U.S. county.

Between January 1, 2019, to June 4, 2021, a total of 1,038,117 user-initiated Breath Alcohol Content (BrAC) recordings were made from 28,452 users. Figure 1 shows the distribution of all BrAC recordings based on the United States county where the reading was obtained, revealing at least some measurements in nearly every state and more than 10,000 measurements in multiple geographically disparate regions around the US. Over a median of 34 days (Interquartile Range [IQR], 1–282 days and total range 1–886 days), each individual made a median of 8 (IQR, 3–25) BrAC recordings. Figure 2 displays the total number of BrAC recordings made on each calendar week, with a shaded red area illustrating where there were SIP orders in place in at least one U.S. county. The mean BrAC level was 0.0437 g/dL ± 0.0635 g/dL, and the median BrAC level was 0.013 g/dL (IQR, 0.0–0.07 g/dL). The minimum BrAC recording was 0.0 g/dL, and the maximum recording was 0.5 g/dL. After restricting the analysis to include only the maximum BrAC reading for each user each day, a total of 574,896 BrAC observations remained.

**Figure 3:**
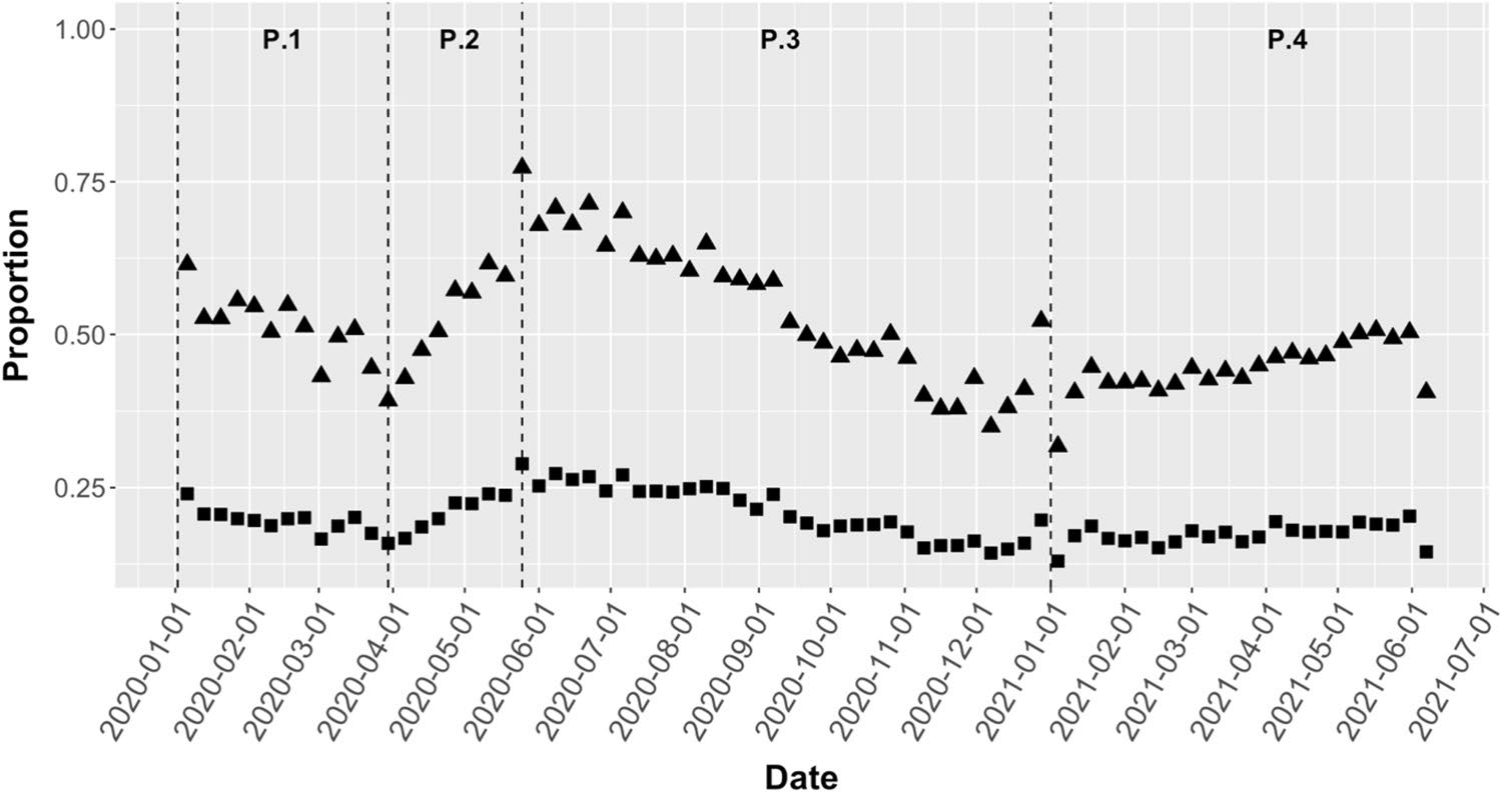
Weekly BrAC proportions between January 1, 2020, and June 4, 2021. The triangles in the figure represent the proportion of BrAC recordings detecting any alcohol consumption (> 0 g/dL) to the weekly proportion of BrAC recordings revealing no alcohol (0 g/dL); and the squares represent the weekly proportion of BrAC recordings at or above the legal driving limit (≥ 0.08 g/dL) to the proportion under the legal driving limit (BrAC recordings < 0.08 g/dL). Four distinct time periods emerged (P.1–P.4) following the beginning of the SARS-CoV-2 outbreak were used for our analysis and are represented between dashed lines. The dates for each time period are summarized in Table 1.

Two comparisons were conducted for each calendar week between January 1, 2019, and June 4, 2021, and plotted: the first compared the proportion of BrAC recordings detecting any alcohol consumption (> 0 g/dL) to the proportion of BrAC recordings revealing no alcohol (0 g/dL); and the second compared the proportion of BrAC recordings at or above the legal driving limit (≥ 0.08 g/dL) to the proportion under the legal driving limit (BrAC recordings < 0.08 g/dL). Figure 3 shows these two proportions plotted over time with four distinct predetermined time periods: January 1, 2020, to March 30, 2020 (Period 1, which included dates prior to US SARS-CoV-2 cases and before broad implementation of shelter-in-place orders); March 30, 2020, to May 25, 2020 (Period 2, which reflected the calendar dates where 25% or more of total counties with SIP orders were enacted); May 25, 2020, to January 1, 2021 (Period 3, which follows until the start of the next calendar year); and January 1, 2021, to June 4, 2021 (Period 4, which encompasses the remaining reads in 2021). The triangles in that figure show the weekly proportion of BrAC recordings revealing evidence of any alcohol consumption, and the squares represent the proportion that was above the legal driving limit—for both, a pattern of an initial decline, followed by a steep rise, followed by a slow decline and with a final slow rise can be visualized.

Whereas detection of any alcohol became progressively less common in Period 1, there was a steep and statistically significant rise in detection in Period 2, a statistically non-significant decline in Period 3, followed by a less steep, but again statistically significant, rise in alcohol consumption during period 4 (Table 1). A similar pattern occurred when comparing the proportion of users consuming alcohol above the legal driving limit compared to those below the driving limit (Table 2).

**Table 1:** Estimated average weekly change in BrAC detection of alcohol (clustered by individuals to account for within-person repeated measures) during SARS-CoV-2 pandemic.

	Start date	Stop date	Avg weekly change	95% CI	P value
Period 1	1/1/20	3/30/20	− 0.0048	− 0.0090, − 0.0006	0.025
Period 2	3/30/20	5/25/20	0.0224	0.0168, 0.0280	< 0.001
Period 3	5/25/20	1/1/21	− 0.0028	− 0.0057, 0.0	0.051
Period 4	1/1/21	6/4/21	0.0035	0.0003, 0.0068	0.033

**Table 2:** Estimated average change in weekly BrAC proportion of recordings ≥ 0.08 g/dL clustered by individuals to account for within-person repeated measures) during the SARS-CoV-2 pandemic.

	Start date	Stop date	Avg weekly change	95% CI	P value
Period 1	1/1/20	3/30/20	− 0.0034	− 0.0058, − 0.0011	0.004
Period 2	3/30/20	5/25/20	0.0116	0.0086, 0.0146	< 0.001
Period 3	5/25/20	1/1/21	− 0.0017	− 0.0035, 0.0001	0.057
Period 4	1/1/21	6/4/21	0.0008	− 0.0011, 0.0028	0.04

**Figure 4:**
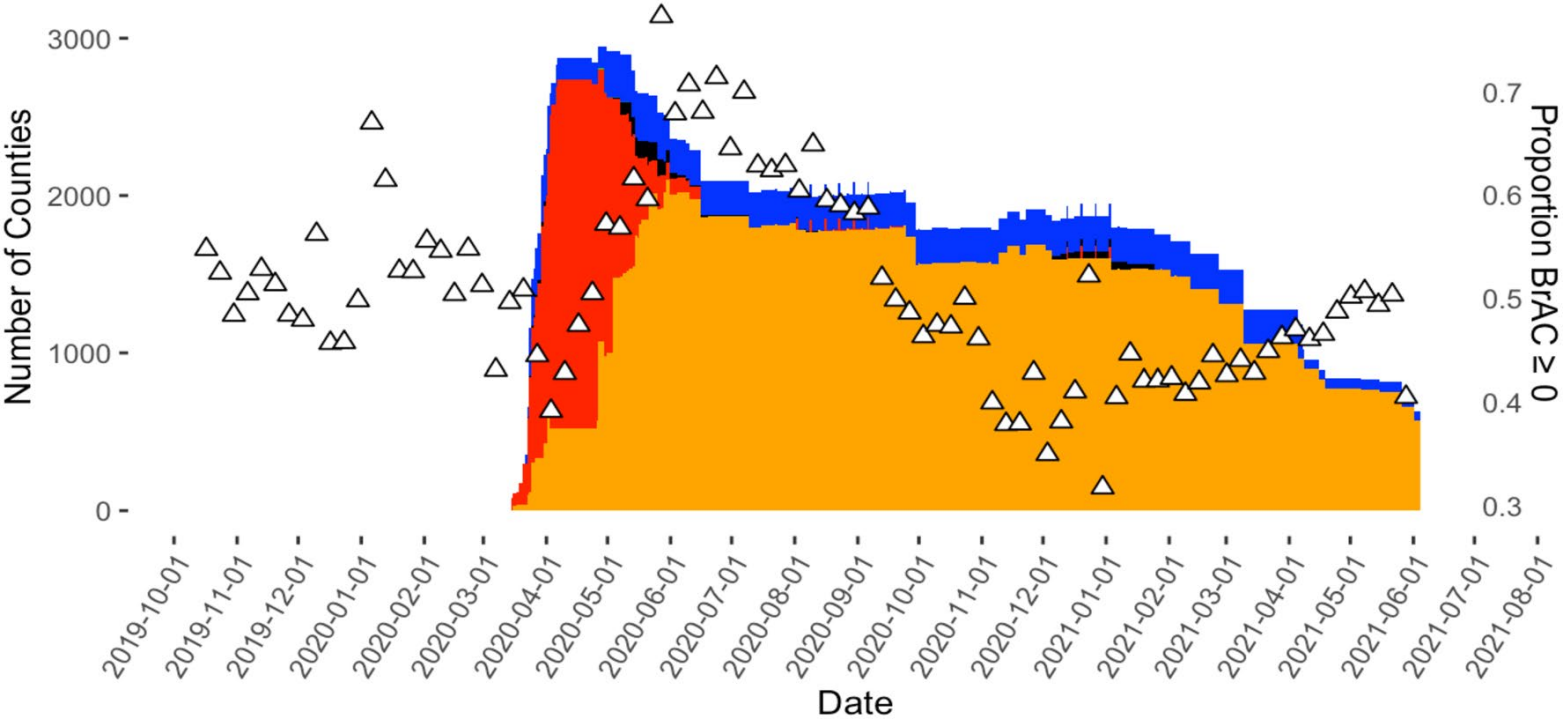
Distribution of Shelter-In-Place orders compared to the weekly proportion of BrAC measurements > 0 g/dL. The open triangles represent the proportion of Breath Alcohol Concentration (BrAC) measurements above 0 g/dL to the proportion of measurements equal to 0 g/dL. The histogram represents the number of U.S. counties with the following Shelter-In-Place orders: red denotes “mandatory for all individuals”; blue denotes “mandatory only for at-risk individuals in the jurisdiction”; black denotes “mandatory only for all individuals in certain areas of the jurisdiction”; orange denotes “advisory/recommendation”; green denotes “mandatory only for at-risk individuals in certain areas of the jurisdiction.

The proportion of users with measurements above 0 g/dL compared to the proportion equal to 0 g/dL plotted against national changes in shelter-in-place orders in Fig. 4. As detailed in the Figure Legend, the different colors represent various gradations of the severity of the SIP orders in different numbers of counties over time. Overall, 2217 US counties implemented at least 1 day of mandatory shelter-in-place for all individuals orders during the study period. From April 17, 2020 to April 23, 2020 all 2217 of these counties had enacted these orders on the same calendar day.

A mutually adjusted logistic model clustered by participant was used to compare the effects of mandatory for all individuals shelter-in-place orders on our within-period drinking proportions. Among counties with SIP orders, there was a declining odds of either any alcohol consumption (Table 3) or alcohol consumption exceeding the legal driving limit (Table 4) for every progressive week in the first time period, and increasing odds per week of similar measurements of alcohol consumption in the time second time period. However, these patterns over the time periods were not statistically significantly different when comparing counties with and without shelter-in-place orders (Tables 3 and 4).

**Table 3:** Estimated relationship between the presence or absence of shelter-in-place order (SIP) on the odds of alcohol consumption for every progressive week within each time period detected by the breathalyzers after accounting for repeated measures within individuals, changes in SIP orders over time, and the day of week and calendar month and year.

		Period 1	Period 2	Period 3	Period 4
Odds ratio	SIP orders	0.95* (CI 0.93–0.98)	1.07* (1.04–1.10)	0.99 (0.98–1.00)	0.99 (0.98–1.00)
	No SIP orders	0.98 (CI 0.91–1.02)	1.03 (0.98–1.09)	1.00 (0.98–1.01)	0.99 (0.96–1.01)
P value for heterogeneity		0.05	0.24	0.71	0.70

*Represents a statically significant value (p < 0.05) within the (SIP or no SIP) location.

P values for heterogeneity refer to statistical significance testing between groups.

**Table 4:** Estimated relationship between the presence or absence of shelter-in-place order (SIP) on the odds of a breath alcohol measurement > 0.08% every progressive week within each time period detected by the breathalyzers after accounting for repeated measures within individuals, changes in SIP orders over time, and the day of week and calendar month and year.

		Period 1	Period 2	Period 3	Period 4
Odds ratio	SIP orders	0.97* (CI 0.95–0.99)	1.03* (1.02–1.07)	1.00 (0.99–1.01)	0.99 (0.98–1.00)
	No SIP orders	0.97* (CI 0.95–0.99)	1.04* (1.00–1.08)	1.00 (0.98–1.01)	0.99 (0.97–1.01)
P value for heterogeneity		0.97	0.96	0.99	0.94

*Represents a statically significant value (p < 0.05) within the (SIP or no SIP) location.

P values for heterogeneity refer to statistical significance testing between groups.

## Discussion

Alcohol consumption varied as the COVID-19 Pandemic and related SIP orders took place. Initially, there was a decline in alcohol consumption just as the global pandemic began, followed by a steep rise as pervasive disease and commercial shutdowns occurred, lasting approximately 2 months. While the pandemic and SIP orders persisted, alcohol consumption then slowly declined over the ensuing 6 months, after which, starting in the beginning of 2021, it steadily increased again. As examinations of mandatory SIP orders by specific location did not track with changes in alcohol consumption, it appears these were indicative of general patterns of behavior independent of government-mandated restrictions.

Within our analysis, we sought to describe two different types of drinking behaviors. First to examine the consumption of any alcohol, which we defined as the proportion of BrAC recordings greater than 0. The second behavior we sought to capture was drinking to exceed the US legal BrAC driving limit of 0.08%. Given that this value is a nationally recognized value that impairs function that can result in punitive legal consequences and is known to be associated with multiple health and societal adverse consequences, this was the highest BrAC examined as an outcome in the current analysis. However, the current analyses did not examine higher BrAC levels indicative of acute alcohol toxicity per se, and future work may focus on such outcomes.

Although the exact reasons for the alcohol consumption patterns observed cannot be definitively determined from the data available, speculation can be reasonably informed by the well-established phenomena affecting the public during the contemporaneous time periods. During the first few months of the pandemic (Period 1) associated with a decline in alcohol use, the virus was just beginning to enter the United States and there was a general uncertainty among the population about what changes to expect. It is possible such uncertainty and a sense of the need to be adaptable and prepared resulted in less time drinking alcohol, or perhaps the initial and abrupt closure of establishments that served and sold alcohol led to fewer opportunities to drink, particularly for people who consumed alcohol while socializing with others. Then as the pandemic continued (Period 2) and employment and other activities began to be placed on hold throughout the country, many people found themselves at home with more free time and certainly less need to drive to work or in-person business establishments. Other research has also indicated growing anxiety during this time^2–4^, which may have also contributed to the rise in alcohol use during this time period. Around the time that mandatory shelter-in-place orders were lifted (period 3), activities began to return to normal for many, such as opportunities (or even obligations) to leave the house, itself shown to be associated with a reduction in alcohol consumption^11^. While period 4 may be the closest representation to a return to baseline, it is all the more concerning that this period yet reveals a steady increase in alcohol consumption. Although our data most directly simply describe drinking trends within our specific sample, our current methodology does not allow for a definitive exploration into the underlying causes of the observed phenomena. We therefore cannot exclude the possibility that other truly causal factors not sufficiently addressed in our presumed explanation were operative.

Previous studies have agreed that there has been a general increase in alcohol consumption during the COVID-19 Pandemic. The use of Bluetooth breathalyzers provides an objective and longitudinal dataset that may provide new insight into the actual trends in alcohol use during the pandemic. Indeed, these analyses did not rely on self-report, the data was collected repeatedly in real-time, and the information was passively obtained (not requiring any special engagement or effort on the part of the > 25,000 individuals using these devices in the course of their normal lives). Prior studies relying on general surveys and self-report of alcohol consumption during the early months of the COVID-19 pandemic revealed findings consistent with ours mainly in regard to the rise in alcohol consumption during the first few months of the pandemic; however, these studies generally lacked granular detail of these patterns and did not include long-term follow up beyond May 2020^9,11^. Our findings also generally fit with rises in the retail sales of alcohol^17^ and alcohol-related deaths^16^.

We did not detect any statistically significant differences related to shelter-in-place orders. While similar patterns of alcohol consumption over time were observed among counties without shelter-in-place orders, we cannot exclude the possibility that our study had insufficient power to detect real differences. Interestingly a study done by Killgore et al. found a positive association between the length of SIP orders and an increase in mean Alcohol Use Disorders Identification Test (AUDIT) score, a questionnaire to measure hazardous alcohol consumption^22^. Our current study focused more on the presence versus absence of SIP orders, and, taken together, these data may suggest that the duration of the SIP orders may be a more potent determinant of harmful alcohol consumption.

Several limitations of the current study should be acknowledged. While we benefited from seamless use of the devices among people using them for their own purposes, they were not following a uniform protocol that assured serial and regular assessments over time. However, presumably individuals would use these devices only when drinking alcohol, and this limitation would not negate the validity of changes in alcohol concentration within the same people over time. Clearly, individuals who decide to purchase and use a Bluetooth-enabled breathalyzer will be different than the general population. As such, it may not be appropriate to generalize drinking habits within this sample group to the general population. Additionally, our limited knowledge of the characteristics and motivation for using these devices in our population could introduce potential cofounders into our analysis. However, we recently published a study revealing that the alcohol consumption patterns of such individuals match times and events when individuals in the general population are expected to consume more alcohol and are indicative of a higher risk of a cardiac outcome in the general population now established as a consequence of alcohol consumption^23^.

Because these data were not collected according to a prespecified study protocol (but instead reflected actual real-world use among thousands of breathalyzer users), the number of BrAC measurements was variable throughout the study. Although our analyses focused on proportions of measurements meeting certain criteria rather than absolute numbers as our outcome of interest, we acknowledge that variability in measurements, such as less measurements in the latter half of period 3, may have introduced bias. While we can be confident of changes over time and the general events related to COVID-19 at those times, we can only infer potential reasons why patterns of alcohol consumption changed over the time periods described. While the strength of our dataset is that it allows for multiple real-time and objective measures, our analysis lacks other measures during these time periods that may influence an individual’s desire to drink, including job status, social isolation, and as well as subjective measures like mood and stress. Future work to incorporate additional measures into this data may help characterize and explain the alcohol use trends we see.

As the SARS-CoV-2 virus continues to spread globally, it is important to define and study the unintended consequences associated with the COVID-19 pandemic. By using Bluetooth-enabled breathalyzer recordings, the objective data suggests that the relationship between alcohol consumption and the COVID-19 pandemic is more complicated than just a mere increase in consumption as other studies have suggested. More broadly, these relationships may offer insight into drinking patterns among large populations related to various stressful or disruptive events.

## Methods

### Users

We analyzed 1,038,117 unique BrAC observations from 28,452 unique users, collected using a commercially available smart-breathalyzer between January 1, 2019, and June 4, 2021. These devices were purchased by participants for their own personal use, and measurements were made on their own accord. Data was provided to researchers at the University of California, San Francisco through a data-sharing agreement. This analysis was conducted using a de-identified dataset and, as users were not contacted or known by the research team, there was no option to opt their data out of the study. In addition to BrAC measurements, this dataset consisted of timestamps and geolocation. The proportion of BrAC measurements made in each county in the United States was calculated and plotted.

BrAC observations were then organized by user, and duplicate measurements were removed. Measurements were then restricted to one BrAC reading per person per day and filtered to the maximum BrAC value by that individual on that given day. This was done under the assumption that an individual may take multiple measurements throughout a day, especially during times of heavy drinking, which in return could bias data towards the lower ends of drinking. Additionally, by restricting to the highest measurement of the day, it would most closely represent a person’s number of drinks in a day and thus that day’s drinking pattern.

After constraining the dataset to only include the max BrAC recording taken for each specific user on each calendar day, 574,896 BrAC observations remained for analyses. The geolocations provided with each BrAC reading were cross-referenced with the shelter-in-place orders database to categorize the nature of shelter-in-place orders at the time of the measurement.

### Shelter-in-place orders

Data for shelter-in-place orders by county was collected from the Centers for Disease Control (CDC) and Prevention website^1^. In this dataset, 3233 counties were tracked through the collection of official documents and press releases from official government websites between March 15, 2020, and August 15, 2021, where each county was assigned one of the following orders each calendar day: “Mandatory for all individuals,” “Advisory/Recommended,” “Mandatory only for at-risk individuals in the jurisdiction,” “Mandatory only for all individuals in certain areas of the jurisdiction,” “Mandatory only for at-risk individuals in certain areas of the jurisdiction,” or “No order for individuals to stay home.” Given the absence of any evidence of a shelter-in-place order for a given location during a particular time, such areas during those time periods were designated as “No order for individuals to stay at home.”

### Data analyses

Two distinct weekly proportions of BrAC measurements were calculated. The first compared the proportion of BrAC recordings detecting any alcohol consumption (> 0 g/dL) to the proportion of BrAC recordings revealing no alcohol (0 g/dL); and the second compared the proportion of BrAC recordings at or above the legal driving limit (≥ 0.08 g/dL) to the proportion under the legal driving limit (BrAC recordings < 0.08 g/dL). These proportions were then plotted by calendar week.

Four time periods based on the distribution of “mandatory for all shelter in place” orders and calendar year were defined and used for the analysis of those proportions. Period 1 spanned from the start of 2020 until the week following the enactment of 25% (554+ counties) of the number of counties with mandatory SIP orders placed (January 1, 2020–March 30, 2020). Period 2 encompassed the consecutive weeks with over 25% implementation of mandatory SIP orders (March 30, 2020–May 25, 2020). Period 3 (May 25, 2020–January 1, 2021) extended up to the beginning of the subsequent year, while Period 4 (January 1, 2021–June 4, 2021) included all available measurements in 2021.

To estimate the change in trends in weekly BrAC levels between these four distinct time periods, a linear regression model with robust standard errors clustered by individual (to take repeated measures within individuals into account) was employed. To estimate average BrAC levels within each predetermined period time-dependent indicators for each period were included in the model. Additionally, linear splines were utilized by calendar date to estimate and analyze the average weekly change of BrAC proportions within each time period.

To determine if there were differences in drinking patterns for individuals in counties that had mandatory SIP orders versus no SIP orders, individuals were first divided among those who were ever present in a county with such an order versus those who were never located in such a county. Then, a mutually adjusted logistics model was used to compare the effects of SIP (versus the absence of such orders) within each time period. To account for repeated measures within individuals, the model clustered by individual and adjusted for the day of the week, calendar month, and year. F-tests were used to assess for evidence of period-specific differences in level and trend, and the model was tested for heterogeneity.

Statistical analyses were conducted using R version 4.2.0 (R Project for Statistical Computing, Vienna, Austria). Two-tailed p values < 0.05 were considered statistically significant.

### Ethical parameters

All methods were carried out in accordance with relevant guidelines and regulations and experiments were approved by the University of California, San Francisco Institutional Review Board (approval number #18-26720). This was an analysis of a de-identified dataset using information gleaned from commercially-available devices. The informed consent was waived and was approved by the University of California, San Francisco Institutional Review Board.

## Data Availability

The data that support the findings of this study are available from BACtrack (San Francisco, CA) but restrictions apply to the availability of these data, which were used under license for the current study, and so are not publicly available. Data are however available from the author, Gregory M. Marcus, upon reasonable request and permission of BACtrack.
